# Intramuscular DNA Vaccination of Juvenile Carp against Spring Viremia of Carp Virus Induces Full Protection and Establishes a Virus-Specific B and T Cell Response

**DOI:** 10.3389/fimmu.2017.01340

**Published:** 2017-10-24

**Authors:** Carmen W. E. Embregts, Dimitri Rigaudeau, Tomáš Veselý, Dagmar Pokorová, Niels Lorenzen, Jules Petit, Armel Houel, Malte Dauber, Heike Schütze, Pierre Boudinot, Geert F. Wiegertjes, Maria Forlenza

**Affiliations:** ^1^Cell Biology and Immunology Group, Department of Animal Sciences, Wageningen University, Wageningen, Netherlands; ^2^INRA, Infectiologie Expérimentale Rongeurs Poissons, Université Paris-Saclay, Jouy-en-Josas, France; ^3^Veterinary Research Institute, Brno, Czechia; ^4^National Veterinary Institute, DTU, Lyngby, Denmark; ^5^INRA, Virologie et Immunologie Moléculaires, Université Paris-Saclay, Jouy-en-Josas, France; ^6^Friedrich-Loeffler-Institut, Federal Research Institute for Animal Health, Institute for Infectiology, Insel Riems, Germany

**Keywords:** DNA vaccination, spring viremia of carp virus, T cells, B cells, rhabdovirus

## Abstract

Although spring viremia of carp virus (SVCV) can cause high mortalities in common carp, a commercial vaccine is not available for worldwide use. Here, we report a DNA vaccine based on the expression of the SVCV glycoprotein (G) which, when injected in the muscle even at a single low dose of 0.1 µg DNA/g of fish, confers up to 100% protection against a subsequent bath challenge with SVCV. Importantly, to best validate vaccine efficacy, we also optimized a reliable bath challenge model closely mimicking a natural infection, based on a prolonged exposure of carp to SVCV at 15°C. Using this optimized bath challenge, we showed a strong age-dependent susceptibility of carp to SVCV, with high susceptibility at young age (3 months) and a full resistance at 9 months. We visualized local expression of the G protein and associated early inflammatory response by immunohistochemistry and described changes in the gene expression of pro-inflammatory cytokines, chemokines, and antiviral genes in the muscle of vaccinated fish. Adaptive immune responses were investigated by analyzing neutralizing titers against SVCV in the serum of vaccinated fish and the *in vitro* proliferation capacity of peripheral SVCV-specific T cells. We show significantly higher serum neutralizing titers and the presence of SVCV-specific T cells in the blood of vaccinated fish, which proliferated upon stimulation with SVCV. Altogether, this is the first study reporting on a protective DNA vaccine against SVCV in carp and the first to provide a detailed characterization of local innate as well as systemic adaptive immune responses elicited upon DNA vaccination that suggest a role not only of B cells but also of T cells in the protection conferred by the SVCV-G DNA vaccine.

## Introduction

The strong increase in the consumption or use of fish products over the last decades has been the result of the ongoing intensification of the whole aquaculture sector. This intensification, however, has led to the increasing incidence of infectious diseases for which no effective vaccines are yet available. In response to this, an increasing emphasis has been placed on the development of experimental vaccines for fish and the investigation of fish immune responses after vaccination (1–5). Besides the various vaccination strategies currently employed in the aquaculture sector, including intraperitoneal injection or immersion vaccination, experimental DNA vaccination has been reported for a broad range of fish viruses and was shown to be especially effective against fish rhabdoviruses when administered by intramuscular injection. Furthermore, a major step forwards toward the commercialization of DNA vaccines for fish was made in April 2016 when the European Medicine Agency gave, for the first time, a positive advice toward their use in Europe by granting marketing authorization for the CLYNAV DNA vaccine against salmon pancreatic disease (6). Effective DNA vaccines against fish rhabdoviruses are reported against viral hemorrhagic septicemia virus (VHSV) in rainbow trout (*Oncorhynchus mykiss*) (7), Japanese flounder (*Paralichthys olivaceus*) (8), turbot (*Scophthalmus maximus*) (9), and Pacific herring (*Clupea pallasii*) (10); against infectious hematopoietic necrosis virus (IHNV) in Chinook salmon (*Oncorhynchus tshawytscha*), sockeye salmon (*Oncorhynchus nerka*) (11), and rainbow trout (12); and against hirame rhabdovirus in Japanese flounder (13). Furthermore, combined DNA vaccination against VHSV and IHNV was shown to induce protection against both viruses in rainbow trout (14). In all these successful vaccines, the DNA plasmid coded for the rhabdovirus glycoprotein (G).

Carp is the most cultured fish species worldwide, and the ornamental variant, koi carp, are very high value fish (15). Their production, however, is threatened by several bacterial and viral diseases. Among those, spring viramia of carp (SVC) is caused by SVC Virus (SVCV), a cytopathic virus belonging to the genus *Vesiculovirus* of the *Rhabdoviridae* family causing an acute systemic infection in several cyprinid species (16). SVC is widespread throughout Europe and has been diagnosed in other parts of the world including the US (17, 18) and China (19). SVCV virions contain one linear negative-sense single-stranded RNA molecule that codes for five structural proteins. The G protein of SVCV, which is the only viral protein present on the virion surface and forms trimeric peplomers, binds to cellular receptors to induce viral endocytosis and is the target of protective neutralizing antibodies (16, 20, 21). Outbreaks of SVCV cause severe losses in carp production, especially during spring, and mainly affect juvenile carp for which mortality rates can be as high as 90% (16).

To date, DNA vaccines against SVCV have been shown to induce only limited protection (22, 23), much lower than reported for G protein-based DNA vaccines against other fish rhabdoviruses including IHNV and VHSV (24). Recently, a recombinant *Lactococcus plantarum* expressing both the SVCV G protein and the koi herpes virus (cyprinid herpesvirus 3) Open Reading Frame 25 was reported to induce moderate protection against both viruses (25). While the aforementioned studies show the potential of G protein-based vaccines against SVCV, strong protection against SVCV viral challenge has not been reported thus far for any experimental vaccine. Furthermore, the lack of an optimized challenge model, closely resembling the natural route of SVCV infection, hampered vaccine evaluation as most studies report the use of intraperitoneal injections as the preferred challenge route.

Characterization of local and systemic immune responses after DNA vaccination against rhabdoviruses has been performed for various fish species. A rapid induction of type-I interferons and interferon-stimulated genes (ISGs) such as *mx, isg15*, and *viperin* was reported in multiple studies (14, 26–28) and can therefore be considered one of the hallmarks of the rapid, non-specific, antiviral response induced by DNA vaccination. The rapid local upregulation of *mx* was suggested to be the main determinant for the observed cross-protection against IHNV after DNA vaccination using plasmids encoding the G protein of either snakehead rhabdovirus or SVCV (29). The expression of two microRNAs, which are strongly induced in rainbow trout either infected with VHSV or DNA vaccinated against VHSV, is apparently correlated with upregulation of type-I *ifns, ifn*γ, and *mx* genes and may play a role in the modulation of the response (30).

DNA vaccination of fish also induces an adaptive immune response. In trout, it was found that protection against VHSV was essentially based on the presence of neutralizing antibodies, which are detectable at 2–3 weeks after vaccination (14). T cell responses have also been studied, and it was shown that VHSV-G DNA vaccination induced a specific T cells response that comprised the same public response as induced by the virus itself (31). Furthermore, PBLs isolated from VHSV-G DNA vaccinated trout specifically killed VHSV-infected but not IHNV-infected target cells (32). To date, detailed characterization of the immune response of carp after DNA vaccination against SVCV has not been performed.

In this study, we report the establishment of a reliable SVCV bath challenge for common carp. Using this challenge model we first examined the age-related susceptibility of carp to SVCV, to estimate the best timing of vaccination and the window of high disease susceptibility. We found that carp were most susceptible at the age of 3 months and were fully resistant at 9 months. Having assessed the importance of protecting carp at a young age, we vaccinated 3-month-old carp at 20°C, with two doses of an intramuscularly (i.m.) injected SVCV-G protein-based DNA vaccine, and showed the ability of the vaccine to induce 95–100% protection against SVCV, even when administered at a low dose (0.1 µg/g fish). To investigate the rapid local immune response induced after DNA vaccination, we raised an antibody against the SVCV G protein and used it together with leukocyte-specific antibodies to examine the expression of the G protein in the muscle and the recruitment of leukocytes to the site of injection. By real-time quantitative PCR (RT-qPCR) we characterized the expression of a panel of immune genes related to the innate and adaptive response. Through analysis of virus-specific humoral and cellular responses we investigated the neutralizing activity in serum of vaccinated fish and the presence of antigen-specific T-cells by performing an *in vitro* proliferation assay.

Altogether, this is the first study reporting (1) a reliable SVCV bath challenge model, (2) age-related susceptibility of carp to SVCV, (3) the optimization of a G protein-based DNA vaccine conferring full protection against SVCV, and (4) the detailed characterization of local as well as systemic humoral and cellular immune responses triggered upon DNA vaccination in carp. Overall, this report contributes to the understanding of the protective mechanisms triggered by DNA vaccination in carp and will play an essential role in the design of future SVCV-G-based vaccination strategies in carp, the species representing the biggest fish production in the global aquaculture.

## Materials and Methods

### Animals

European common carp (*Cyprinus carpio carpio*) R3 × R8 were used that originated from a cross between the Hungarian R8 strain and the Polish R3 strain (33). In this study, we will refer to carp as the European common carp subspecies, unless stated otherwise. Carp were bred in the Aquatic Research Facility (ARC) of University’s animal facility, Carus at Wageningen University, the Netherlands and were either kept at the local facility, transported to the Veterinary Research Institute (VRI, Brno, Czech Republic), or to the Institut National de la Recherche Agronomique (INRA, Paris, France) for viral challenge experiments. Carp were raised at 20–23°C in recirculating UV-treated water and fed pelleted carp food (Skretting, Nutreco) twice daily.

### Virus

The reference SVCV strain VR-1390, isolate stock of the INRA laboratory (34, 35), was propagated in epithelioma papulosum cyprinid (EPC) cells grown in Glasgow’s modified Eagle’s medium (GMEM)–25 mM HEPES (Eurobio) supplemented with 10% fetal calf serum (FCS; Eurobio), 1% tryptose phosphate broth (Eurobio), 2 mM l-glutamine (PAA), 100 µg/mL penicillin (Biovalley), and 100 µg/mL streptomycin (Biovalley) in the absence of CO_2_. The SVCV CAPM V 539 strain (36) was propagated in common carp brain (CCB) cells at 27°C or in EPC cells at 20°C. EPCs were grown in MEM medium (Gibco) supplemented with 10% FCS, 2 mM l-glutamine, 100 µg/mL penicillin, and 100 µg/mL streptomycin in the presence of CO_2_. For CCBs, the same medium was supplemented with 3.5 g/L d-glucose and 1% non-essential amino acids (Gibco). Virus titers were determined by the method of Reed and Muench (37) and were given as plaque-forming units (pfu).

### Characterization and Validation of a Monoclonal Antibody against the SVCV G Protein

Spring viremia of carp virus of the Fijian strain (35, 38) was used to raise antibodies against the SVCV G protein in mice. Before immunization, female Balb/c mice were given a tolerizing treatment to reduce reactions to cell proteins, as described before (39). After such treatment, mice were immunized with 1.4 × 10^8^ pfu of concentrated and purified SVCV in complete Freund’s adjuvant. The same viral dose was given 4 weeks later in incomplete Freund’s adjuvant, followed by two more doses in the following 85 days. Three days after the last immunization mouse spleen cells were isolated and fused with Sp2/0 myeloma cells. Obtained hybridoma supernatants were screened through Western blot and immunofluorescence of SVCV infected and pcDNA3-SVCV-G transfected cells. For Western blot analysis, purified SVCV and lysates from non-infected EPC cells were resolved on 15% SDS-PAGE gels. Proteins were transferred to nitrocellulose membranes and incubated with hybridoma supernatants (1:10) or with an available anti-SVCV rabbit polyclonal serum (1:2,000) as positive control. Western blot development was performed as described before (38, 39). For immunofluorescence analysis, EPCs were infected with an MOI of 1 for 24 h at 20°C. In parallel, EPCs were seeded in 6-well plates, transfected with 2 µg of pcDNA3-SVCV-G or pcDNA3 using 7 µL of FuGENE HD (Promega) following the manufacturer’s guidelines. EPC was imaged 48 h after transfection. Infected or transfected cells were fixed with 4% PFA for 15 min at 4°C and incubated with hybridoma supernatant from selected clone 13C10c (1:150) for 1 h and with goat-anti-mouse-RPE (BioLegend, 1:500) for 30 min at room temperature. A counterstaining with DAPI (Thermo Scientific) was included to stain the cells nuclei. Images were acquired with an EVOS fl LED fluorescence microscope (Advanced Microscopy Group).

### Optimization of SVCV Challenge

For all viral challenges the water temperature was gradually lowered from 20 to 15°C at a rate of 1–2°C/day. Optimization of the SVCV challenge was performed at INRA, using the VR-1390 strain. Three-month-old carp (*n* = 20 per group, 2–4 g) were challenged by immersion, i.m. or intraperitoneal (i.p.) injection. For immersion challenge, carp were exposed to a dilution of SVCV-containing EPC supernatants (8 × 10^6^ pfu/mL) for either 3 or 48 h in a volume of 4 L (biomass 15 g/L). A control group (*n* = 20) was exposed to uninfected EPC cell culture supernatant by immersion and was treated similarly. Fish were also challenged by injection; they were anesthetized using 0.3 g/L tricaine methane sulfonate (TMS, Crescent Research Chemicals) before i.m. injection of 1,000 pfu/fish or i.p injection of 5,000 or 10,000 pfu/fish. Water quality monitoring included pH (8) and oxygen (>7 mg/L). Fish were observed daily, and moribund fish were removed from the tanks.

Age-related sensitivity to SVCV infection was investigated at VRI, using the SVCV CAMP V 539 strain and juvenile carp between 3 and 9 months (20 g). R3 × R8 carp, all from the same hatching batch, were raised under the same condition [water flow (15 L/h) pH (7.5–8), oxygen (10–12 mg/L), and N-NH_4_ (<0.2 mg/L)] up until 3 months of age. At that time, a subgroup of fish (*n* = 20 per group) was transferred to 100 L tanks having the same water conditions, were acclimatized to the temperature of 15°C and challenged by immersion for 30 h with 8 × 10^6^ pfu/mL. The remaining fish were kept under controlled water quality condition up until 7 and 9 months, and then challenged following the same protocol. Fish were observed daily, and moribund fish were removed from the tanks.

### DNA Vaccination

The pcDNA3-SVCV-G DNA vaccine was prepared as described previously (32) using the G-protein sequence of the CAPM V 539 strain Jaroslavicky 97 (accession number: KU934300). All vaccinations were performed at 20°C. Carp of 3 months (*n* = 10 per group, 1.5–2 g) were assigned to either the pcDNA3 empty plasmid group (negative control) or the pcDNA3-SVCV-G group (vaccine). Carp were anesthetized in 0.3 g/L TMS and vaccinated i.m. in the epaxial muscle, below the dorsal fin with 1 µg (first experiment) or 0.1 µg (second experiment) of DNA plasmid/g of fish in 10 µL PBS.

For assessment of vaccine efficacy, carp were challenged 2.5–3 months after vaccination, at 15°C, using the optimized challenge method, and survival was monitored over a period of 3–5 weeks. In parallel, mid kidneys were isolated from a subgroup of carp (*n* = 6) to confirm SVCV infection through analysis of *svcv n* gene expression. The mid kidney is one of the organs in which virus replication occurs, even early after infection and can be used for virus re-isolation of monitoring of viral infection (40).

### RNA Isolation and cDNA Synthesis

For gene expression analysis of the local response at the injection site, carp injected with the low plasmid dose (0.1 µg/g fish) were sacrificed at 3 and 5 days postinjection (dpi). Carp were euthanized in 0.6 g/L TMS and bled through the caudal vein. Muscle at the injection site was isolated, immediately snap frozen in liquid nitrogen, and stored at −80 until further processing. Total RNA was isolated from muscle and mid kidney tissue using the RNeasy Mini Kit (Qiagen) according to the manufacturer’s instructions including on-column DNase treatment using the RNase-free DNase set (Qiagen). For RNA isolation from muscle tissue, an additional Proteinase-K (Qiagen) treatment was included. RNA concentrations were measured using a Nanodrop-1000, the integrity was verified on a 1% agarose gel and RNA was stored at −80°C until further use. Before cDNA synthesis of 1 µg total RNA, a second DNase treatment was performed using DNAse I, Amplification Grade (Invitrogen). Reverse transcription of the RNA was performed using random primers (300 ng) and Superscript™ III (200 U) First Strand Synthesis Systems for RT-PCR (Invitrogen). cDNA samples were further diluted 25 times in nuclease-free water and stored at −20°C.

### Gene Expression Analysis

Real-time quantitative PCR was performed using a Rotor-Gene™ 6000 (Qiagen). Fluorescence data were analyzed using Rotor-Gene Q series software version 2.3.1. Briefly, 5 µL of 25 times diluted cDNA was mixed with 2 µL of forward and reverse primers (2.1 µM of each primer) and 7 µL of 2× ABsolute qPCR SYBR Green Mix (Thermo Scientific) as detection chemistry. The list of primers can be found in Table 1. The take-off value for each sample and the average reaction efficiencies (*E*) for each primer set were obtained upon comparative quantitation analysis from the Rotor-Gene software (41). The relative expression ratio (*R*) of each sample was calculated according to the Pfaffl method (42) based on the take-off deviation of sample versus each of the unhandled controls at time point 0 h and normalized relative to the *s11* protein of the *40s* subunit as reference gene. For analysis of the *svcv-n* gene during infection with SVCV, the housekeeping gene β*-actin* was used, since it was found to be the most stable under these circumstances.

**Table 1:** Primers used in real-time quantitative PCR.

Primer	FW primer 5′–3′	RV primer 3′–5′	Acc. no.
**Housekeeping genes**			
*40s*	CCGTGGGTGACATCGTTACA	TCAGGACATTGAACCTCACTGTCT	AB012087
β*-actin*	CAACAGGGAAAAGATGACACAGATC	GGGACAGCACAGCCTGGAT	CCACTBA
**SVCV detection**			
*svcv-n*	TGAGGTGAGTGCTGAGGATG	CCATCAGCAAAGTCCCGGTAT	NC_002803
**Cytokines**			
*cxca*	CTGGGATTCCTGACCATTGGT	GTTGGCTCTCTGTTTCAATGCA	AJ421443
*cxcb1*	GGGCAGGTGTTTTTGTGTTGA	AAGAGCGACTTGCGGGTATG	AB082985
*cxcb2*	AGGCAGGTGCTTCTGTGCTGACA	TTCATGCATTTCCGCTCTGCGCT	JN104598
*il1β*	AAGGAGGCCAGTGGCTCTGT	CCTGAAGAAGAGGAGGAGGCTGTCA	AJ245635
*il6a*	CAGATAGCGGACGGAGGGGC	GCGGGTCTCTTCGTGTCTT	KC858890
*il6b*	GGCGTATGAAGGAGTGAGGG	TGCTCCTCTCTCGGTCAGAT	KC858889
*tnfα*	GCTGTCTGCTTCACGCTCAA	CCTTGGAAGTGACATTTGCTTTT	AJ311800 and AJ311801
**Transcription factors**			
*stat1*	GAGACGGAGGAATCACC	GGATGTCTGGGTAAAGGTAG	KJ782028
**Interferons**			
*ifn*γ*2a/2b*	CGATCAAGGAAGATGACCCAGTC	GTTGCTTCTCTGTAGACACGCTTC	AM168523
*ifn*φ*1*	GCACGTATACAAAGATGAACC	TGATCCAAGGTCAAGACAAG	GQ168341
*ifn*φ*2*	TTGGTGTAAAAAAGGCAACC	GCTGCTTTCTCGTCATAATAC	JN741616
**Interferon-stimulated genes**			
*mx1*	ACAATTTGCGGTCTTTGAGA	CCCTGCCATTTCTCTTCG	cypCar_00015892
*mx2*	GCTTACGGTCTCTGGGG	TGGTTTCATCTTTAGTTCTTATCATC	cypCar_00029512
*vip2*	CTGTCGGACACATCAGC	TCAATGGGCAAGACGAAA	cypCar_00024055
*pkr3*	CACGGTGTTTGAAAAGAGC	GACTGGGTCTCAGCATTC	cypCar_00039221
*isg15.2*	AGTGTTCGTCAAGAATGAGG	CCTCGCAGACGGAAAAC	cypCar_00039111
**Adaptive immune genes**			
*igm*	CACAAGGCGGGAAATGAAGA	GGAGGCACTATATCAACAGCA	AB004105
*igt1*	AAAGTGAAGGATGAAAGTGT	TGGTAACAGTGGGCTTATT	AB598367
*igt2*	GATTCTACTGGGT8CTTCAC	GACATCACTCAACTC8TTCT	AB598368
*zap70*	GGAACAAGCCATCATTAGCC	GTCGTCTCTCACCCTCCTG	Scaf 2523 and 63374

### Immunohistochemistry

To visualize the expression of the SVCV G protein at the site of injection, carp (3–4 g) were i.m. injected with 20 µg of pcDNA3 (empty plasmid) or pcDNA3-SVCV-G plasmid in 20 µL PBS containing 0.01% green tattoo dye (Eickemeyer). Carp were sacrificed 7 and 14 days dpi and bled through the caudal vein before collecting muscle tissue at the site of injection.

Cryosections (5 µm) from muscle sections were stained with specific antibodies as described before (43). For the detection of the SVCV-G protein, slides were stained with anti-SVCV-G clone 13C10c diluted 1:150 and alkaline phosphatase (AP) conjugated goat-anti-mouse (Dako) (1:200). Development was performed using AP substrate [4.5 µL/mL nitro-blue-tetrazolium (Roche Applied Science) and 3.5 µL/mL 5′-bromo-4′-chloro-3′indolyl phosphatase (Roche Applied Science) in AP buffer (0.1 M Tris–Cl, 0.1 M NaCl, 0.05 M MgCl_2_) until sufficient staining was observed]. For the detection of neutrophilic granulocytes, slides were stained with the TCLBE8 antibody [1:50 (43, 44)] and GAM-AP (1:200, Dako). Development was performed as described earlier. Tissue morphology was examined using a Hemacolor^®^ eosin-azur staining (Merck Millipore). Pictures were made using a Leica DM6 microscope and analyzed using the Leica LAS X program.

### Neutralization Assay

Sera from fish injected with 1 µg/g of either pcDNA3 or pcDNA3-SVCV-G plasmid were collected 2.5–3 months after vaccination and were used to quantify neutralizing titers. Blood was drained from the caudal vein and was let to clot at 4°C overnight. Serum extraction was performed by centrifugation at 2,000 *g* for 10 min, and the obtained supernatant was centrifuged at 10,000 *g* for 20 min. Serum was heat-treated at 56°C for 30 min, aliquoted and frozen at −20°C before use in titration assays. To determine SVCV neutralization titers, carp serum was mixed with an equal volume of GMEM 2% FCS, DEAE 1× (Sigma), containing carp complement (standardized serum pool from naïve carp, diluted 1:80), and incubated 4 h at 20°C with 2.1 × 10^2^ pfu SVCV (strain VR-1390). One hundred microliters of the mixture were then transferred onto confluent EPC monolayers in 24 wells and incubated for 1 h at 14°C. After this step, melted methylcellulose was added to the wells, and plates were kept at 24°C for 3 days. After 3 days, plates were fixed with 4% formaldehyde and stained with crystal violet to reveal viral plaques. Controls included non-infected wells and wells infected with SVCV without preincubation with carp serum. Plaques were counted manually and an upper threshold on the number of counted plaques, indicating a fully infected well, was set at 100 plaques.

### *In Vitro* Antigen-Specific B and T Cell Proliferation Assays

Carp were vaccinated i.m. with 1 µg DNA/g of fish of pcDNA3-SVCV-G DNA vaccine. The same amount of pcDNA3 plasmid was used as injection control. PBLs were isolated 3 months after vaccination, separated on Ficoll Paque (GE Healthcare) as described previously (45, 46), and stained with carboxyfluorescein succinimidyl ester (CFSE) (47). Part of the CFSE-labeled cells (2 × 10^7^ cells/mL) was transferred to round-bottom 96-well culture plates (Corning) and stimulated with SVCV (MOI of 25) or, as a control, with equivalent volumes of CCB-conditioned culture medium, for 2 h at 27°C. Cells were then seeded in 48-well plates (Corning) at a density of 2 × 10^6^/well in Advanced DMEM/F-12 (Life Technologies) supplemented with 2 mM l-glutamine, 100 U/mL penicillin G, 50 mg/mL streptomycin sulfate, 1% FCS, and 10^−5^ M 2-mercaptoethanol (Sigma). In parallel, a fraction of both SVCV-treated or mock-treated cells was incubated in the presence of recombinant carp interferon gamma (Ifnγ2, 100 ng/mL) or interleukin-10b (rIl10b, 0.25 U/mL) since we reported before that these cytokines are able to enhance carp leukocytes pro-inflammatory activities (48) and (antigen-specific) proliferation of memory cells (47, 49–51). Cells were incubated for 6 days at 27°C in the presence of 5% CO_2_.

Proliferation of Zap70^+^ T cells and Igm^+^ B cells was analyzed by flow cytometry using a cross-reactive antibody for the pan T cell marker Zap70 (47) and the mouse monoclonal antibody (WCI12) against carp Igm (52). Cells were collected after 6 days, washed once with PBS and incubated for 30 min at RT with Zombie Red™ fixable viability dye (1:1,000 in PBS, BioLegend). For the subsequent analysis of T cell proliferation, cells were washed once with FACS buffer [0.5% BSA (Roche), 0.01% NaN_3_ in PBS] and were fixed and permeabilized using the Cytofix/Cytoperm Kit (BD Biosciences) according to the manufacturer’s protocol. Cells were washed once in FACS buffer and incubated for 30 min on ice in 35 µL of anti-Zap70 rabbit mAb (99F2; Cell Signaling, 1:100). After two washes with FACS buffer, cells were incubated in 35 µL of PE-conjugated goat-anti-rabbit IgG (Santa Cruz, 1:100). For B cell proliferation, cells were washed and incubated as described earlier with WCI12 mAb (1:100) and goat-anti-mouse IgG-PE (Santa Cruz, 1:100). After subsequent washes, cells were analyzed on a FACS CantoA (BD Biosciences), and data were analyzed using FlowJo V10 (BD Biosciences). Proliferation of B or T cells was analyzed by first gating on the live cells (Zombie Red™, negative) subsequently for the specific cell staining (either WCI12 for Igm^+^ B cells or Zap70^+^ for total T cells, PE channel), and finally for the CFSE staining (visible in the FITC channel) of the identified population. The intensity of the CFSE staining at day 0 was used to set the thresholds for proliferation. At day 6, a decrease in CFSE fluorescence intensity was indicative of cell proliferation. Obtained percentages of proliferation in each treated group were corrected with their respective conditioned medium-only control by subtraction.

### Statistical Analysis

Statistical analysis was performed for gene expression, neutralization assay, and proliferation assay data. All data were analyzed using SPSS Software 22 (IBM). For gene expression data, relative expression ratios R were transformed [LN(R)], and significant differences (*p* < 0.05) between pcDNA3 and pcDNA3-SVCV-G at the indicated time point were determined by a one-way ANOVA followed by the Tukey *post hoc* test. Plaque count of the neutralization assay and the percentages of proliferating Zap70^+^ T cells from the proliferation assay were analyzed using a one-way ANOVA.

## Results

### Prolonged Exposure of Juvenile Carp to SVCV at 15°C Results in a Reliable Bath Challenge Model of Infection

To evaluate vaccine efficacy, the establishment of a reliable and reproducible infection model is of utmost importance. To the best of our knowledge, the first optimization of an SVCV bath challenge was described in 1978 by Ahne (53) exposing 25–30 g carp fish to 2 × 10^3^ pfu for 2 h at 13°C. Subsequent reports describe similar procedures. In our hands, however, such protocol resulted in high variability between replicate challenges and generally low mortality rates (data not shown). Therefore, we first established a reliable and robust (bath) infection model closely mimicking the natural route of virus infection. We initially investigated the effect of virus dose, time of exposure and infection route on infection efficacy.

**Figure 1:**
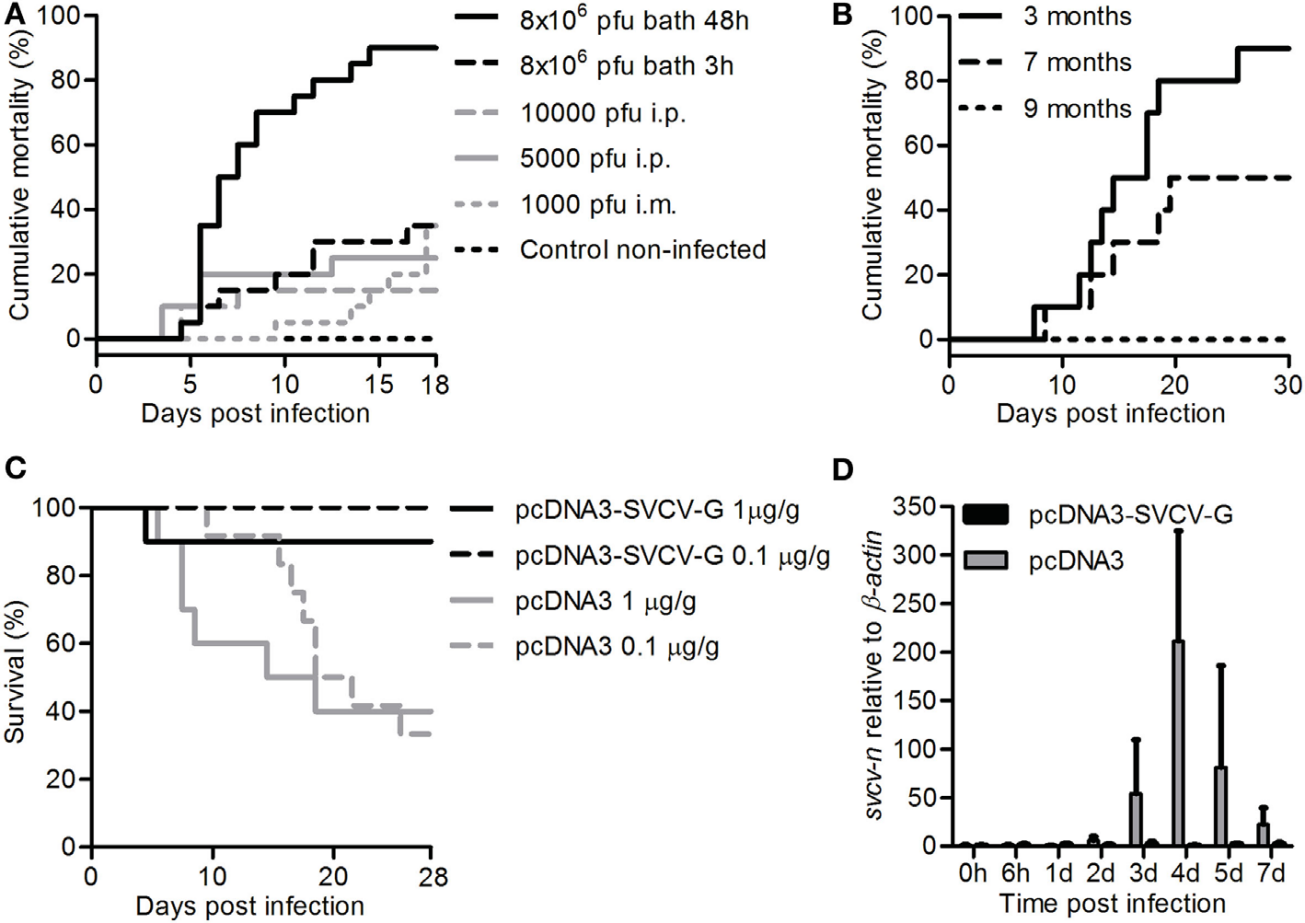
Establishment of a bath challenge for spring viremia of carp virus (SVCV) and validation of a protective DNA vaccine. **(A)** Carp (*n* = 20/group) of 3 months were acclimatized to a temperature of 15°C, exposed to the indicated doses of SVCV (VR-1390 strain), for the indicated time, and mortality was recorded. **(B)** Carp (*n* = 10) were challenged at 3, 7, or 9 months of age by bath for 30 h at 15°C using 8 × 10^6^ pfu/mL of the CAPM V 539 SVCV strain, and mortality was recorded. **(C)** Three-month-old carp (*n* = 10/group) were i.m. injected with 1 µg/g of fish of either pcDNA3 or pcDNA3-SVCV-G and challenged 2.5 months postvaccination, for 48 h at 15°C using 8 × 10^6^ pfu/mL SVCV (VR-1390). In a subsequent experiment, fish were vaccinated with 0.1 µg/g of fish of the same plasmids and challenged as described previously. Survival was monitored over a period of 4 weeks. **(D)** Carp were treated as in panel **(C)** using 1 µg/g of fish of DNA plasmid, and upon challenge mid kidneys were isolated at the indicated time points. *svcv-n* gene expression was analyzed by real-time quantitative PCR. Gene expression was normalized relative to β-actin as a housekeeping gene and expressed relative to the unhandled controls collected at time point 0 h. Data are shown as average + SD of *n* = 6 fish. Abbreviation: d, day.

Carp of 3 months were exposed to SVCV (VR-1390 strain) by i.m. or i.p. injection as well as by bath at a temperature of 15°C. Challenge by i.m. or i.p. injection resulted in mortality rates of 35 and 15–25%, respectively, independent of the viral dose used (Figure 1A). Similar mortality rates were observed after bath challenge for 3 h at 8 × 10^6^ pfu/mL. Bath challenge using the same viral load but with an exposure time of 48 h resulted in high mortality (up to 90%) within 15 days. Altogether, the data indicate that prolonged exposure of juvenile carp to SVCV, in a bath challenge at 15°C, results in high mortality rates.

Using the optimized bath challenge method, we next investigated the effect of age on SVCV susceptibility to look for the best time window to later on investigate vaccine efficacy. To test the robustness of the bath challenge, we used the closely related SVCV CAMP V 539 strain in an interlaboratory experimental setup. For this, 3, 7, or 9-month-old carp were exposed to 8 × 10^6^ pfu/mL of the CAPM V 539 SVCV strain by bath for 30 h at 15°C. A strong age-dependent effect on disease susceptibility was observed (Figure 1B). While again up to 90% mortality could be observed in 3-month-old carp, mortality rates rapidly decreases to 50% in 7-month-old fish, and only 0–20% mortality was observed in 9-month-old carp. Altogether, we developed a reliable bath challenge with optimized temperature, viral load, and exposure time. Using this optimized bath challenge, we show that susceptibility of carp to SVCV is age dependent, and that the optimized challenge is reliable independent on the viral strain used.

### Intramuscular Vaccination Using a G Protein-Based DNA Vaccine Induces 95–100% Protection against SVCV, Even When Administered at a Low Dose

Optimization of the bath challenge model showed that carp are most susceptible to SVCV at young age (between 3 and 6 months). Therefore, to test the efficiency of DNA vaccination, carp were vaccinated at an age of 3 months and subsequently challenged at an age of 6 months (2.5–3 months after vaccination). This assured that we were still within the age window of high susceptibility to SVCV (<6 months), but also that the strong non-specific, type-I IFN-dependent protection induced by DNA vaccination in fish would have faded (24).

Carp were vaccinated at 20°C with 1 µg of pcDNA3-SVCV-G/g fish and challenged 2.5 months later at 15°C using the aforementioned bath challenge. While survival in the pcDNA3-injected group was around 40%, the pcDNA3-SVCV-G-vaccinated group had 90% survival (Figure 1C), which is equivalent to a relative percent survival (RPS) of 83.3. To investigate whether a lower dose of vaccine would be sufficient to protect against SVCV, in a subsequent experiment carp were vaccinated with 0.1 µg of the vaccine per gram of fish and challenged as described. In this experiment, full protection (100 RPS) in the vaccinated group was observed 2.5 months after vaccination (Figure 1C). In the group vaccinated with the high vaccine dose, the development of an SVCV infection was verified by analysis of the SVCV N gene expression in mid kidneys of vaccinated and non-vaccinated fish after SVCV challenge. *Svcv n* gene expression was detected from 2 days postinfection onward in non-vaccinated fish (pcDNA3) but not in vaccinated fish (pcDNA3-SVCV-G) (Figure 1D), confirming that vaccination with the pcDNA3-SVCV-G vaccine strongly suppressed SVCV infection. No expression of the N gene was observed in non-challenged fish (data not shown). Altogether, the results indicate that the pcDNA3-SVCV-G vaccine, even at a low dose of 0.1 µg/g fish, is able to confer protection against SVCV for a period long enough to cover the age window during which carp are most susceptible to SVCV.

### SVCV-G Protein Is Expressed in the Muscle after DNA Vaccination and Triggers a Strong Local Immune Response

**Figure 2:**
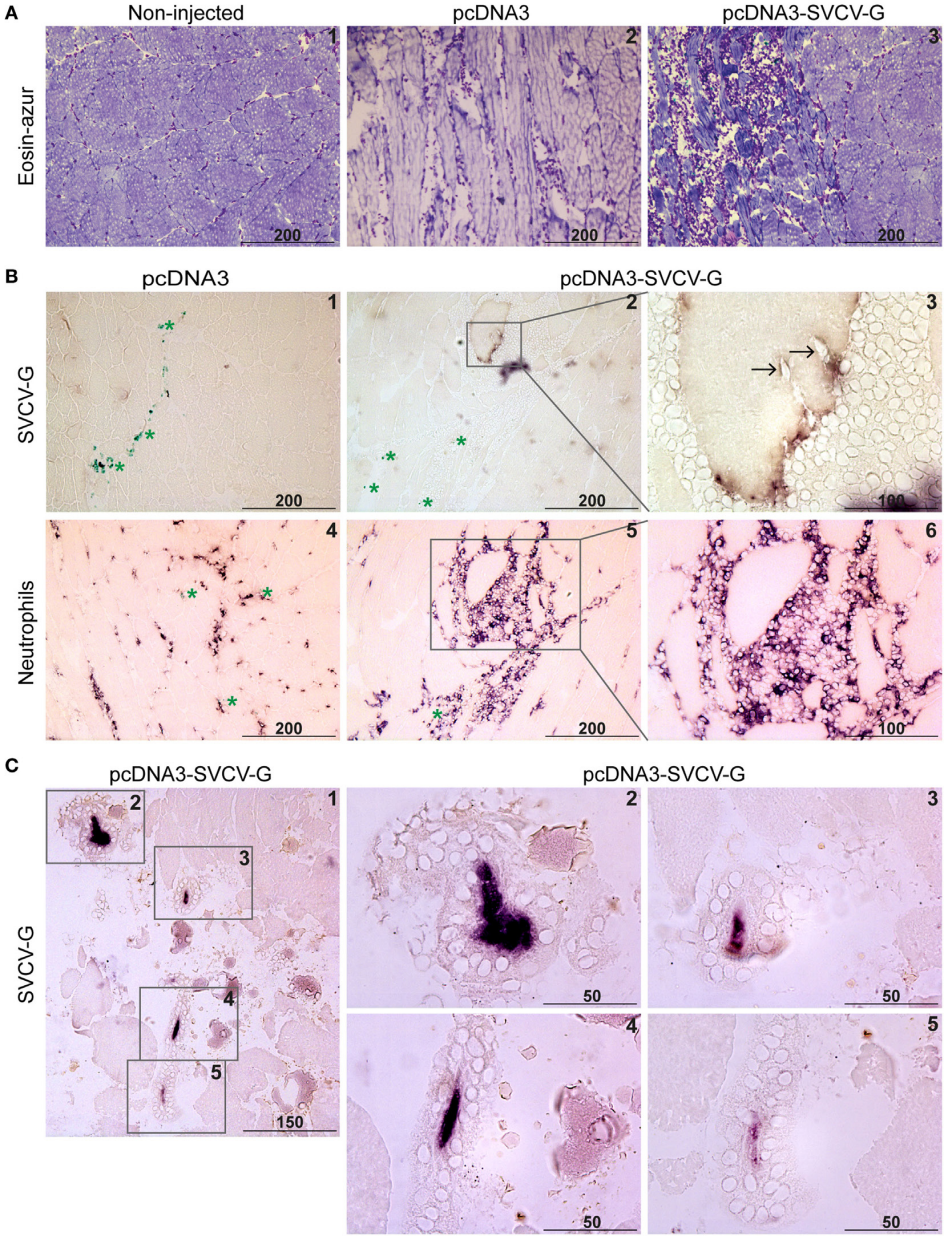
Immunohistochemical analysis of G-protein expression and leukocyte recruitment after DNA vaccination. Carp were injected with 20 µg of pcDNA3 or pcDNA3-spring viremia of carp virus (SVCV)-G. **(A)** Muscle was isolated at the site of injection at 7 days postinjection (dpi), and cryosections (5 µm) were stained with Hemacolor^®^ (eosin-azur) to visualize leukocyte recruitment in non-injected (A.1), pcDNA3 injected (A.2), or pcDNA3-SVCV-G injected (A.3) tissue. **(B)** Muscle at the site of injection was isolated 7 dpi from carp injected with pcDNA3 (B.1) or pcDNA3-SVCV-G (B.2–3) and stained with anti-SVCV-G antibody (clone 13C10c). Green asterisks in panel (B.1) indicate ink particles along the needle path. Brown color in panel (B.2) indicates G-protein expression. Note that the G protein-expressing cell is detached from the rest of the tissue and surrounded by leukocytes. A magnification of panel (B.2) shows the G-protein reactivity [(B.3), purple/brown color] on the myocyte surface and the presence of leukocytes around and infiltrating (black arrows) the myocyte. A consecutive slide of the tissue at the same time point was stained using an antibody specific for carp neutrophilic granulocytes (B.4–6); the inset in panel (B.5) identifies the same G protein-expressing cell as in panels (B.2,B.3). **(C)** Muscle at the site of injection isolated 14 dpi was stained as described in panels (B.1–3); (C.1) overview of the muscle area in which four G protein-expressing myocytes could be detected; (C.2–5) higher magnification of the areas indicated by the insets in panel (C.1); note the condensation of the G-protein staining (purple color) and the presence of leukocytes surrounding the G protein-expressing myocytes. Scale bars indicate distance (in µm).

**Figure 3:**
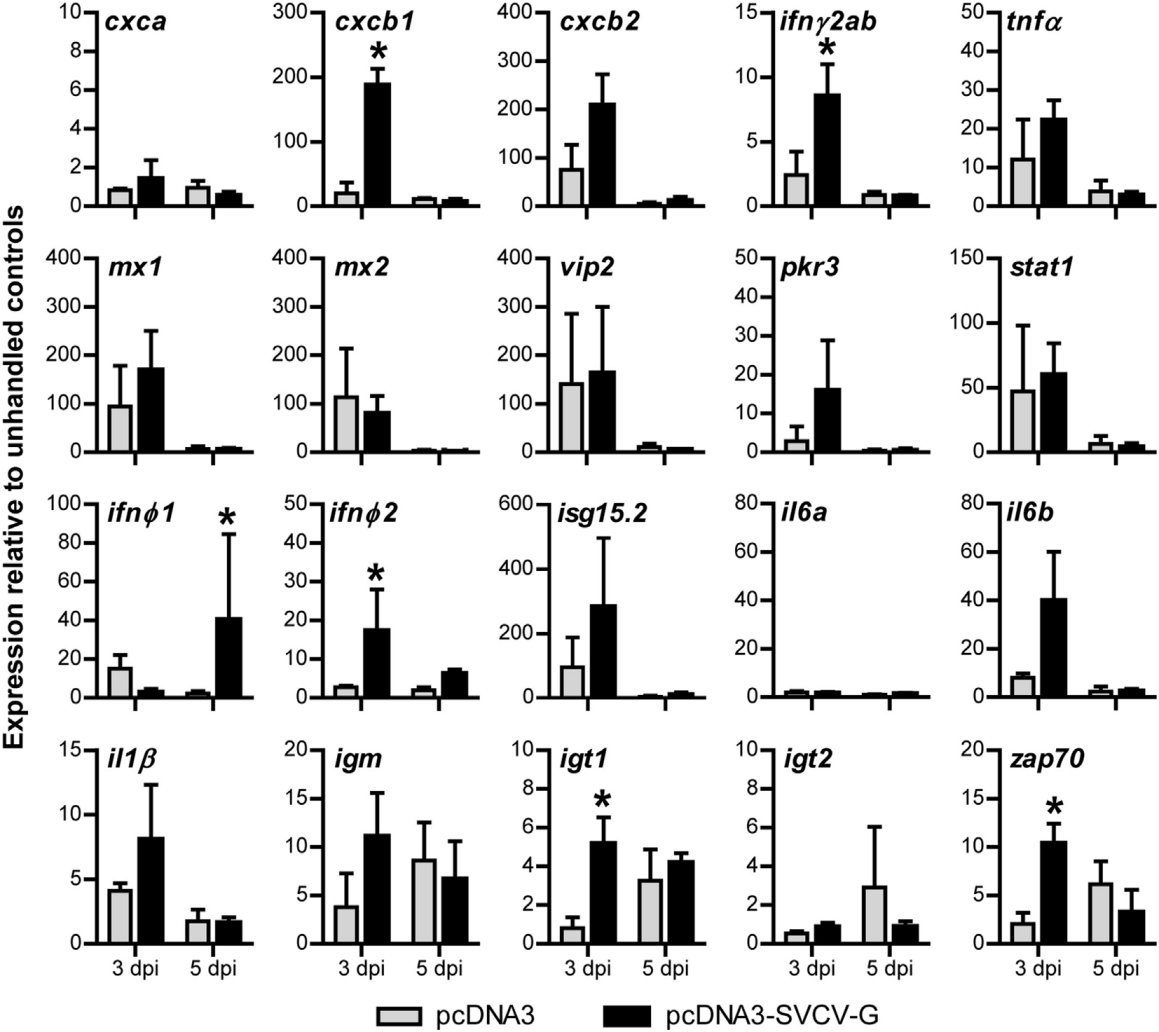
Gene expression analysis of the local immune response after i.m. DNA vaccination against spring viremia of carp virus (SVCV). Carp were injected with 0.1 µg/g of either pcDNA3 or pcDNA3-SVCV-G. Muscle tissue at the site of injection was excised at 3 and 5 days postinjection (dpi). Expression of the indicated immune-relevant genes was normalized against the housekeeping gene *s11* of the ribosomal subunit 40S and expressed relative to the unhandled control at time point 0 h. Asterisks (*) indicate significant differences (*p* < 0.05) between the pcDNA3 and pcDNA3-SVCV-G group at the respective time point as assessed by one-way ANOVA, followed by a Tukey *post hoc* test. Bars indicate average and SD of *n* = 3 fish per time point.

To visualize the expression of the SVCV G protein after i.m. administration, and to investigate the tissue damage as well as the local immune response, carp were injected with 20 µg of either pcDNA3 or pcDNA3-SVCV-G plasmid. Muscle tissue from the site of injection was excised at 7 and 14 dpi from both groups, and the anti-SVCV-G antibody (characterized and validated in Figure S1 in Supplementary Material) was used to visualize G-protein expression. To visualize the influx of leukocytes after vaccination a Hemacolor^®^ eosin-azur staining was used, as well as specific antibody staining for neutrophilic granulocytes and macrophages. A strong influx of leukocytes was observed at 7 dpi in muscle tissue of both pcDNA3 injected (Figure 2A, 2) and pcDNA3-SVCV-G vaccinated group (Figure 2A, 3), indicating that the influx is strongly damage- and inflammation-driven, mainly caused by the injection itself. Myocytes expressing the viral G protein on their cell membrane were detected at 7 as well as 14 dpi (Figure 2B, 2–3 and Figure 2C). At 7 dpi, myocytes expressing the G protein were found to be surrounded by a large number of leukocytes and were disconnected from neighboring cells. Furthermore, the leukocytes appeared to infiltrate myocytes positive for G-protein expression (Figure 2B, 3, black arrows).

**Figure 4:**
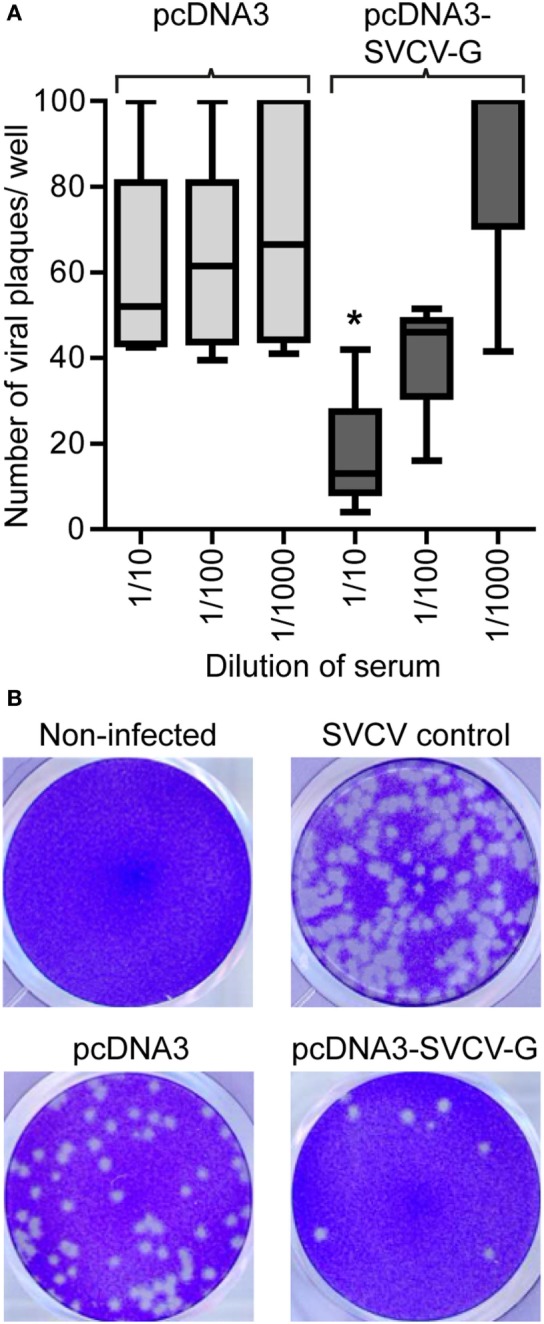
Neutralizing capacity of serum from i.m. DNA vaccinated carp. Serum (*n* = 5/group) was collected from vaccinated [pcDNA3-spring viremia of carp virus (SVCV)-G] and non-vaccinated (pcDNA3) carp 3 months after vaccination and used in a neutralization assay using the SVCV VR-1390 strain. Epithelioma papulosum cyprinid (EPC) cells were fixed and stained with crystal violet after 3 days of infection. **(A)** Quantification of the number of virus plaques per well. Box plots indicate the minimum, maximum, and average plaques count. The upper threshold on the number of counted plaques, indicating a fully infected well, was set at 100 plaques. Asterisk (*) (*p* < 0.05) indicates a significant difference between the pcDNA3 and the pcDNA3-SVCV-G group at the given dilution as assessed by an one-way ANOVA. **(B)** Representative pictures of wells containing non-infected EPCs monolayers (non-infected), EPC infected with SVCV only in the absence of carp serum (SVCV control), SVCV incubated with serum from pcDNA3 injected control fish (pcDNA3) or with serum from pcDNA3-SVCV-G vaccinated fish (pcDNA3-SVCV-G).

To verify the presence of specific leukocytes subtypes in the area surrounding the G protein-expressing cells, an antibody specific to neutrophilic granulocytes was used (Figure 2B, 4–6). An influx of neutrophils was observed at 7 dpi in muscle injected with the pcDNA3 plasmid (Figure 2B, 4) and to a larger extent in the pcDNA3-SVCV-G injected tissue (Figure 2B, 5–6). Moreover, the neutrophil-specific staining revealed that a large proportion of leukocytes surrounding G protein-expressing myocytes are neutrophils. Macrophages were recruited to the site of injection at this time point as well, but in lower numbers than neutrophils (data not shown).

A prominent change in muscle morphology at the injection site was observed at 14 dpi, with a clear deterioration of the muscle tissue. At this time point, the G protein-expressing myocytes were condensed, as indicated by the concentrated G-protein staining, detached from the surrounding myocytes and completely surrounded by leukocytes (Figure 2C).

In conclusion, we found an injection-related inflammation in the muscle. G protein-expressing myocytes were clearly surrounded by large numbers of leukocytes, especially neutrophils. At 14 dpi a complete isolation of the G protein-expressing myocytes from the surrounding muscle tissue was observed, along with cell condensation. Altogether, this suggests that G-protein expression in the tissue leads to a robust response against G protein-expressing cells, which in turn might favor activation of protective mechanisms.

### Intramuscular DNA Vaccination Induces a Rapid Upregulation of Immune-Related Genes at the Site of Injection

Given the high protection conferred by the DNA vaccine plasmid (Figure 1C), and the strong inflammatory response observed locally at the site of injection at 7 and 14 dpi (Figure 2), we next investigated the early local gene expression profile induced by vaccination. A panel of pro-inflammatory cytokines and chemokines, antiviral genes as well as adaptive immune markers, were selected to reveal which pathways were activated before the observed leukocyte recruitment (Figure 2). Carp were injected with 0.1 µg/g plasmid, and muscle tissue at the site of injection was isolated at 3 and 5 dpi for subsequent gene expression analysis (Figure 3).

At 3 dpi, the chemokine *cxcb1* (54, 55) and the cytokines *inf*γ*2ab* and *ifn*φ*2* were specifically upregulated by the injection of pcDNA3-SVCV-G plasmid (vaccine group), but not by the empty plasmid. At 5 dpi, *ifn*φ*1* was also significantly upregulated in the vaccine group. By contrast, in both the pcDNA3 and in the pcDNA3-SVCV-G injected groups, the pro-inflammatory molecules *cxcb2, tnf*α, *il6b*, and *il1*β and the antiviral genes *mx1, mx2, vip2, pkr3, stat1*, and *isg15.2* were all elevated at 3 dpi. The overall upregulation of pro-inflammatory genes confirmed the previous observation (Figure 3) that a local inflammatory response marked by a strong recruitment of leukocytes is triggered in part by the damage caused by the injection and by the plasmid backbone, and in part is specific for the vaccine plasmid. Interestingly, when looking at markers of adaptive immune cells, a vaccine-specific upregulation was observed at 3 dpi for *igt1* and *zap70*, whereas *igm* transcripts were elevated, but not significantly compared with the control group, altogether suggesting that pcDNA3-SVCV-G promoted an early recruitment of B and T cells at the site of injection. At 5 dpi, the increased expression of these markers was no longer specific for the vaccine as transcription was elevated in both the pcDNA3 and pcDNA3-SVCV-G injected group.

Altogether, our results indicate that injection of plasmid DNA in the muscle of carp induces a strong local inflammatory response, that is, in part specific for the vaccine plasmid. Considering the efficacy of the DNA vaccine (Figure 1C), the combined inflammatory response induced by the damage, the plasmid backbone and in part by the SVCV-G protein, might all contribute to provide the optimal conditions for the onset of a specific adaptive response to the SVCV-G protein.

### DNA Vaccination against SVCV Leads to Detectable Virus Neutralizing Titers in the Serum of Vaccinated Fish

To investigate the role of humoral responses induced by i.m. DNA vaccination, we analyzed the neutralizing capacity of serum from vaccinated carp 3 months after vaccination. This is of importance since protection against rhabdoviruses has been shown to strongly rely on the presence of neutralizing antibodies, although they are not always present at detectable titers (24). While no clear inhibition of viral growth was observed when using the serum of pcDNA3 injected carp (non-vaccinated), a significant neutralizing capacity was observed in the serum of pcDNA3-SVCV-G injected carp (vaccinated) (Figures 4A,B). Although only at a serum dilution of 1:10 the number of plaques was significantly different from the controls, a clear decrease in viral plaques was observed also at the 1:100 dilution. This result shows that a single i.m. injection of DNA vaccine encoding for the SVCV G protein, is sufficient to induce virus neutralizing activity in the serum of vaccinated fish, which is most likely mediated by virus neutralizing antibodies.

### DNA Vaccination against SVCV Induces the Formation of SVCV-Specific Zap70^+^ T Cells

We next investigated the presence of SVCV-specific T cells in the blood of vaccinated fish. To this end, we analyzed the proliferative capacity of antigen-specific T cells *in vitro*. PBLs were isolated from carp 3 months after vaccination, stimulated *in vitro* with SVCV alone, or in combination with recombinant Ifnγ2 or Il10b. These two cytokines were selected for their known capacity to promote T cell proliferation in carp. As was reported before, carp Il10b promoted proliferation of “memory” T cells in an *in vitro* study using PBLs and HKLs from carp that survived an infection with the blood-borne parasite *Trypanoplasma borreli* (47). IFNγ enhances antigen-specific T cell proliferation, and in ginbuna crucian carp (*Carassius auratus langsdorfii*) recombinant Ifnγrel was found to enhance numbers of CD4^+^ and CD8α^+^ T cells during allograft rejection (51). Furthermore, a concomitant upregulation of *tbet, ifn*γ*1*, and *ifn*γ*2* was observed upon stimulation of leukocytes from multiple organs with phytohemagglutinin (56). However, the effect of recombinant Il10b and Ifnγ2 on virus-specific T cells generated upon DNA vaccination is still unknown.

**Figure 5:**
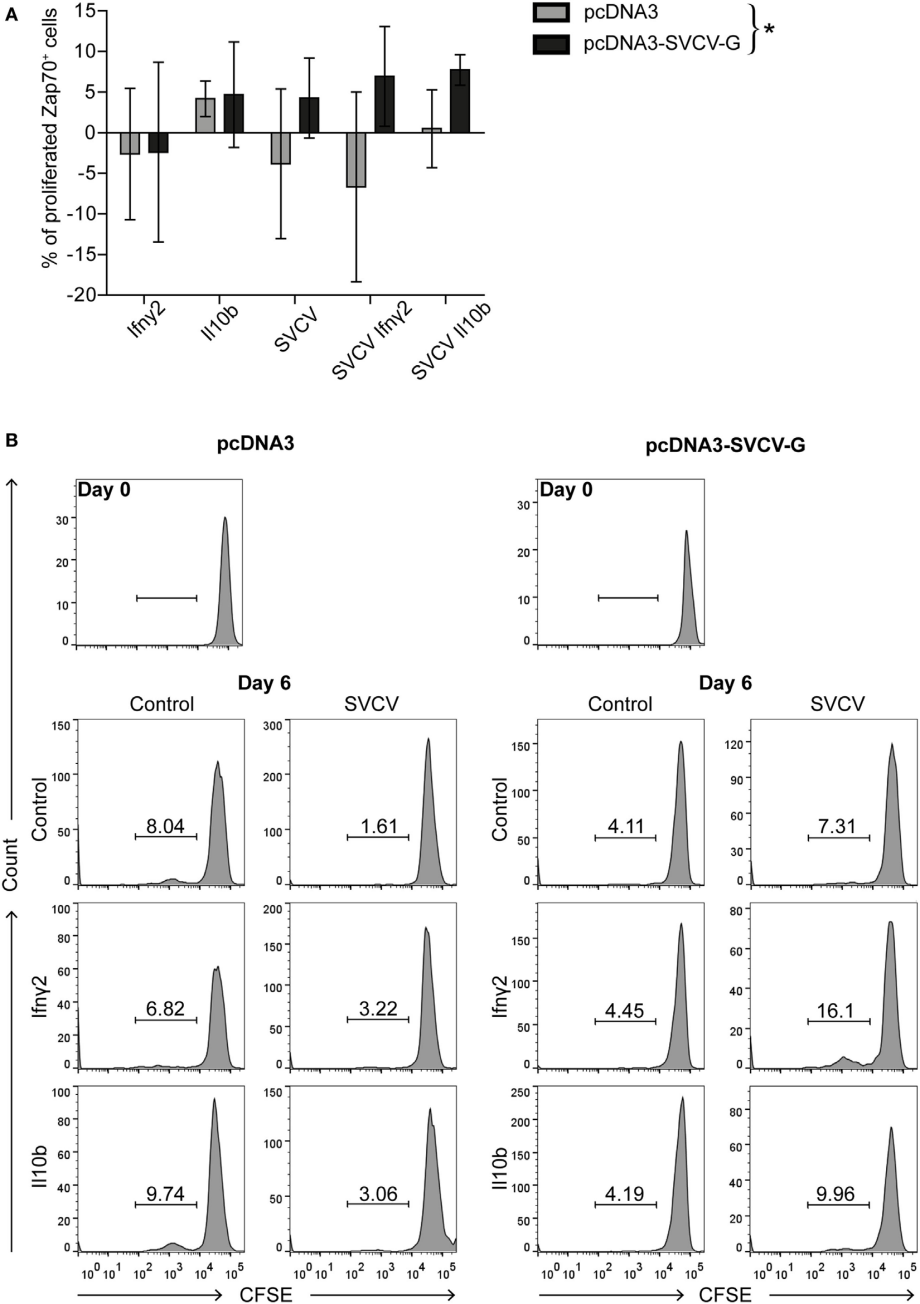
DNA vaccination against spring viremia of carp virus (SVCV) induces the formation of virus-specific T cells. PBLs were isolated 3 months after vaccination from non-vaccinated (pcDNA3) and vaccinated (pcDNA3-SVCV-G) carp. Carboxyfluorescein succinimidyl ester (CFSE)-labeled PBLs were stimulated for 6 days at 27°C with recombinant Ifnγ2 (100 ng/mL) or Il10b (0.25 U/mL) alone, or in combination with SVCV (MOI of 25). As a control, conditioned medium was used. T-cells were identified using a cross-reactive anti-Zap70 antibody, and proliferation was analyzed by flow cytometry. **(A)** Percentages of proliferating Zap70^+^ T cells are expressed relative to the respective conditioned medium control after subtraction of the percentage of proliferating cells in the medium control. For each group, bars represent average and SD of *n* = 7 fish/stimulus, except for the Il10b stimulated samples for which *n* = 3 fish were used. Asterisk (*) indicates a significant differences between the pcDNA3 and pcDNA3-SVCV-G group as assessed by one-way ANOVA. **(B)** Representative histogram plots of CFSE-labeled Zap70^+^ T cells from non-vaccinated (pcDNA3, left panel) and vaccinated (pcDNA3-SVCV-G, right panel) carp of the experiment in panel **(A)**.

Proliferation of Zap70^+^ cells from vaccinated fish was quantified 6 days poststimulation. Proliferation of cells stimulated with recombinant Ifnγ2 or rIl10b alone did not differ between the non-vaccinated (pcDNA3) and vaccinated (pcDNA3-SVCV-G) groups (Figure 5A) however, Il10b, but not Ifnγ2, induced proliferation in both groups. By contrast, stimulation with SVCV induced a proliferative response in the vaccinated group only, and the proliferation was further enhanced by co-stimulation with Ifnγ2 (Figures 5A,B) and, to a lesser extent, by rIl10b. One-way ANOVA analysis showed that the overall proliferative response of Zap70^+^ T cells significantly different between vaccinated and non-vaccinated fish.

A similar approach was used to measure the proliferation of Igm^+^ B cells in PBLs from vaccinated and non-vaccinated fish. This revealed a high proliferative response upon SVCV stimulation in both groups and therefore, no significant differences were observed (data not shown). Altogether, these data indicate that i.m. DNA vaccination against SVCV induces the formation of T cells that recirculate in the blood of vaccinated fish 3 months after vaccination and proliferate when restimulated *in vitro* with SVCV. This proliferation capacity can be further enhanced by Ifnγ2 and to a lesser extent by rIl10b. Whether these T cells are CD4^+^ and/or CD8^+^ will require further investigation.

## Discussion

The efficacy of DNA vaccination to protect carp against SVCV has been controversial, mainly because it is notoriously difficult to set up challenge models with this virus. Here, we used an efficient bath challenge method, which likely mimics the natural infection, to demonstrate that i.m. injection of a plasmid encoding the SVCV G protein affords a high level of protection against SVCV. We did not only investigate the challenge method but also the plasmid dose, the temperature of vaccination and the age of the fish at the time of vaccination, taking into account that carp were found to be most susceptible to SVCV within the first 6 months of age. When investigating the local response induced by i.m. vaccination, our findings reveal a substantial recruitment of neutrophils and macrophages during the first 2 weeks following vaccination. Cells expressing the SVCV-G protein were surrounded by leukocytes, progressively disconnected from the neighboring cells and likely targeted by an immune response. Furthermore, our data show that DNA vaccination leads to the presence of virus-neutralizing activity in the serum of vaccinated fish, which is most likely mediated by neutralizing antibodies, and to the presence of SVCV-specific T cells in the blood of vaccinated fish, which proliferate *in vitro* upon SVCV restimulation. Altogether, these responses are likely responsible for the long-term protection of carp observed 3 months after vaccination upon challenge with SVCV *via* the natural route of infection.

Challenge models that comply with the natural route of infection are of utmost importance for the proper validation of protective effects of experimental vaccines. While intraperitoneal injections are often used because of their ease of standardization, cohabitation, or bath challenges, although typically more difficult to standardize, better resemble the natural route of infection. Here, we report a standardized and reproducible bath challenge procedure for common carp based on prolonged (>30 h) exposure to SVCV (8 × 10^6^ pfu/mL), typically leading to >90% mortality rates in juvenile carp. Previously reported bath challenge procedures for SVCV showed mortality rates of 67–75%, possibly because of the relatively short exposure time (60 min) to the virus and a lower viral load used (5 × 10^3^ pfu/mL) (23). In our hands, such short exposure to the virus led to much lower mortality, typically lower than 30%. While shorter bath challenges of a few hours may be preferred because of practical reasons, longer exposure times generally enhance antigen uptake and possibly vaccine effectiveness (57). Indeed, longer exposure time (>30 h) clearly improved the reproducibility of our bath challenge with SVCV. To test the efficacy of our DNA vaccine against SVCV, we used different temperatures for vaccination (23°C) and for viral challenge (15°C). Higher temperature at vaccination is crucial for a rapid onset of specific immune responses, especially for T cell help and antibody formation, and 23°C falls within the temperature range optimal for carp (58–61). Possibly, vaccination at higher temperature might have contributed to the formation of virus neutralizing antibodies, most likely responsible for the neutralizing activity observed in the serum of vaccinated fish. In agreement, carp kept at 10 and 15°C showed a delay in SVCV clearance from the blood and a delayed development of neutralizing antibodies, when compared with fish kept at 20°C (62). Conversely, a lower temperature is crucial for viral replication and 15°C falls within the temperature range optimal for SVCV, with typical field outbreaks in Spring and associated mortalities occurring at water temperatures between 11–17°C. In fact, carp challenged at 20–22°C typically show no mortalities whereas carp challenged at 10–12°C showed 90% mortality (62, 63). In our hands, carp older than 9 months of age, when kept at 10°C, showed up to 30% mortality when challenged using our optimized challenge method (data not shown), while no mortality was observed when carp of the same age were challenged at 15°C (Figure 1B). Altogether, these data suggest that it is likely advantageous to vaccinate carp at high temperature, to allow for optimal development of protective response. Also in rainbow trout, temperature-dependent differences in the kinetics and immune compartment involved in the response have been described after DNA vaccination against VHSV (64). For example, neutralizing activity was observed in the plasma of fish DNA vaccinated at 15°C whereas negligible or no neutralizing activity was detected in fish vaccinated at 10 and 5°C. In agreement, also the specificity of the protection was shown to be temperature dependent; in fact, only trout kept at 15°C and vaccinated with a DNA plasmid encoding the VHSV-G protein, and not those vaccinated with a plasmid encoding the IHNV-G protein, showed a specific protection against VHSV upon challenged 40 days later. Conversely, trout kept and vaccinated at 10 or 5°C showed a protection against a VHSV challenge independently of whether they were vaccinated with either of the VHSV-G or the IHNV-G DNA plasmids, suggesting a role for non-specific innate immune mechanisms.

We also studied how the “natural” resistance of carp to bath exposure to SVCV increased with age, leading to almost full resistance from 9 months onward, which might be related to the gradual increase of cross-reactive (natural) antibodies developing over time (65). Age-dependent susceptibility to rhabdoviruses has also been reported for rainbow trout and pike to VHSV (66) and for rainbow trout to IHNV (67). In fact, it is a general observation for many other pathogens in fish and other vertebrates (68). This underlines the necessity of vaccinating carp at a young but immune-competent age of 3 months (69) to overcome the age period when they are most sensitive to SVCV (3–6 months). Finally, our data also make clear the need to verify vaccine efficacy within the age period of susceptibility, using the natural route of infection. Our vaccination protocol based on a low dose (0.1–1 µg DNA/g of fish) of pcDNA3-SVCV-G, protected carp against a lethal bath challenge with SVCV 2.5 months after vaccination. Lower doses might be investigated since in rainbow trout, a dose of 0.01 µg DNA/g of fish could protect against IHNV (70). Previous DNA vaccination studies in common carp required higher doses (10–25 µg DNA/g) and/or up to two booster injections (22, 23) but were still less successful in achieving protection against a subsequent SVCV challenge, possibly caused by vaccination at a slightly lower temperature (20°C), or due to difference in the challenge protocol (challenge route, viral strain).

In our study, challenge at >2.5 months after vaccination indicates that the protection is most likely due to the virus specific immune response and based on immune memory. Indeed, the non-specific interferon-induced response that typically arises quickly after DNA vaccination, is generally short-lived (29, 71, 72). The importance of specific immunity in the protection is further supported by the induction of neutralizing antibodies and the presence of virus-specific T cells in the blood of vaccinated fish. “Long-term” (>2.5 months) protective effects of DNA vaccination against SVCV remain to be investigated in carp and would be interesting from an immunological point of view. However, they seem to be of lower practical relevance because of the relatively short window of susceptibility that needs to be covered between the time of immune maturity (>3 months) and development of natural resistance against SVCV (>6–9 months).

Histological analysis of the muscle tissue after injection revealed a strong and rapid influx of leukocytes at the site of injection. This was largely damage- and/or inflammation-driven rather than antigen-specific because it was also seen after injection of the empty plasmid. Yet, the influx of leukocytes into myocytes expressing the SVCV-G protein as well as the time-dependent condensation and degradation of SVCV-G-expressing cells that were completely surrounded by leukocytes, was specific for the SVCV-G injected group. This supports the notion that SVCV-G-expressing cells can be seen by the host as non-self and can trigger both innate as well as SVCV-specific (adaptive) immune responses, at least in part similar to the one triggered by a natural virus infection (24). A similar elimination of myocytes expressing the vaccine antigen was observed in rainbow trout; lymphocytes and macrophages were found in close proximity and infiltrating the G protein-positive myocytes (73). In carp, the influx of leukocytes and the following inflammatory reaction at the site of injection is consistent with the general upregulation of pro-inflammatory genes observed in the groups injected with both, the control or vaccine plasmid. Few genes (*cxcb1, ifn*γ*2, il6b, ifn*φ*1*, and *ifn*φ*2*) among those investigated within our panel were upregulated specifically in the G protein-vaccinated group. Recombinant carp Cxcb was previously shown to stimulate chemotaxis of carp macrophages and granulocytes *in vitro* (74). Also recombinant Il6 has been shown to have synergistic effects on antigen-specific Igm responses of trout, *in vivo*, when co-injected with inactivated infectious pancreatic necrosis virus (75).

The SVCV-G protein-induced upregulation of type-I interferons (*ifn*φ*1* and *ifn*φ*2*) appears an intrinsic property of the G protein since it was also noted for IHNV in rainbow trout (26, 29) and for VHSV in Atlantic salmon (27) and rainbow trout (14, 76, 77). In salmonids, the antiviral interferon response appears to be G protein-specific because *mx* was found upregulated only after i.m vaccination with VHSV-G, but not with VHSV-N (28). In conclusion, although a limited number of genes were specifically upregulated by injection of the pcDNA3-SVCV-G vaccine plasmid, it cannot be excluded that the inflammation caused by the injection-related damage, by the plasmid backbone, and finally by the SVCV-G protein all contribute to the onset of a subsequent specific response toward the G protein.

Of interest, a rapid (7 days) influx of Igm^+^ and Igt^+^ B lymphocytes in the muscle of trout DNA vaccinated with VHSV-G (78), suggests a role not only for innate immune cells but also for B lymphocytes in the early response to vaccination. In carp, SVCV-G-specific upregulation of *zap70* and *igt1* gene expression was noted already at 3 days postvaccination, hinting at a role also in carp of adaptive immune cells in the initial response to DNA vaccination against SVCV. Alternatively, this early wave of B cells may indirectly contribute to fight the virus *via* cytokine production. The importance of B cells in protection against SVCV could be confirmed by the detection of neutralizing activity, most likely mediated by neutralizing antibodies, in the serum of vaccinated, but not control carp. Indeed, neutralizing antibodies have also been reported in early studies following vaccination with inactivated SVCV (53, 62), although this could not be confirmed in a later study with inactivated SVCV (79).

We also examined whether DNA vaccination can induce a T cell response, which would lead to long lasting virus-specific clones. SVCV-G-specific upregulation of the pan T cell marker *zap70* was noted at 3 days after vaccination. Previous studies already suggested a role for cell-mediated immune responses in the protection against SVCV, based on the upregulation of various T-cell markers after SVCV challenge in carp (80) and on a strong lymphocyte proliferation also in the absence of SVCV-specific antibodies in goldfish (22). To gain further insights in the T cell response upon DNA vaccination, we analyzed the proliferative capacity of (Zap70^+^) T cells by stimulating PBLs from vaccinated carp with SVCV *in vitro*. We also examined the potential of two (recombinant) cytokines, Il10b and Ifnγ2, to modulate such proliferative response. Interleukin-10 can have multiple effects on B and T lymphocytes, including regulation of proliferation and differentiation [as reviewed in Ref. (81)], and carp Il10b was shown to promote survival and enhance proliferation of antigen-specific B and T cells (47). Ifnγ2 has multiple effects and in carp was found to enhance antigen-specific responses during *in vitro* stimulation of carp leukocytes and phagocytes (48). We observed an SVCV-specific T cell proliferation in PBLs from vaccinated carp stimulated *in vitro* with SVCV, which could be enhanced by Il10b or Ifnγ2. Despite the large variation in the individual response of PBLs, *in vitro* restimulation with the virus led to an overall significantly higher proliferation of T cells in PBLs isolated from vaccinated fish when compared with the overall T cells proliferation in PBLs from non-vaccinated fish. This suggests that the frequency of peripheral SVCV-specific T cells is higher in vaccinated than in non-vaccinated fish. Although the development of a cell-mediated cytotoxicity response after DNA vaccination against VHSV has been described in rainbow trout (32), in this report we show for the first time virus-specific proliferation of carp T cells *in vitro* after a single low dose injection of DNA vaccine against SVCV. The presence of virus-specific T cells 3 months after vaccination raises the issue of the importance of a T cell-dependent response after the recall: while it is often considered that viral particles provide a perfect matrix of repetitive antigens to induce T cell-independent B cell responses, it is possible that T cell help plays an important role in the immune response of vaccinated fish. The virus-specific T cells present in vaccinated fish may also comprise cytotoxic T cells, of which the contribution to protection would have to be assessed *in vivo*. The development of antibodies against specific subsets of T cells will allow for further investigation of the role of T cells in the establishment of protection against SVCV.

From our data, it appears that already a single low dose of the SVCV-G DNA vaccine is sufficient to trigger both arms of the adaptive immune system. Our data show that DNA vaccination against SVCV induces neutralizing antibodies and suggest that SVCV-specific T cells might contribute to the protection.

Altogether, we for the first time report on a fully protective G protein-based DNA vaccine in carp against SVCV. We also describe age-related susceptibility of carp to SVCV, an optimized bath challenge method, along with the characterization of local as well as systemic protective immune responses after i.m. DNA vaccination against SVCV. Our data provide new insights into the respective implication of B and T cells in the response to the vaccine: an early role for the adaptive immune response and a possible early recruitment of B and T cells to the site of injection. In a later phase of the response we showed the induction of neutralizing antibodies, and the presence of antigen-specific “memory” T cells. This latter finding raises the issue of the relative importance of T cells in the response. Most likely the combination of humoral as well as cell-mediated responses is key to the success of the current DNA vaccine. Given the recent developments in legislation of DNA vaccines for aquaculture species, marked by the approval on the use of the CLYNAV vaccine against pancreatic disease in Atlantic salmon (6), our data might contribute to the increasing need to study DNA vaccines for fish and their underlying mechanisms of protection.

## Ethics Statement

All animals were handled in accordance with good animal practice as defined by the European Union guidelines for the handling of laboratory animals (http://ec.europa.eu/environment/chemicals/lab_animals/home_en.htm). All animal work at INRA was approved by the Ministries of Agriculture and Research, and by Direction of the Veterinary Services of Versailles (authorization number 78-28, project authorization #2707-2016011318282761), as well as fish facilities (authorization number B78-720). Animal work in Wageningen University was approved by the local experimental animal committee (DEC number 2014098). Animal work at VRI was approved by the Branch Commission for Animal Welfare of the Ministry of Agriculture of the Czech Republic (permission No. MZe 1717).

## Author Contributions

CE, MF, JP, DR, TV, GW, and PB contributed to the design of the experiments, acquisition of samples, and analysis of data. PB, GW, and MF acquired funding. NL, DP, MD, HS, and AH contributed with reagents, materials, and analysis tools. CE, MF, GW, NL, and PB wrote the manuscript.

## Conflict of Interest Statement

The authors declare that the research was conducted in the absence of any commercial or financial relationships that could be construed as a potential conflict of interest.
