# Supplementary material for: Intramuscular DNA Vaccination of Juvenile Carp against Spring Viremia of Carp Virus Induces Full Protection and Establishes a Virus-Specific B and T Cell Response
Source: Front Immunol. 2017 Oct 24;8:1340. doi: 10.3389/fimmu.2017.01340 (PMC5660689; doi:10.3389/fimmu.2017.01340)
Supplement: Supplementary file 1 [file Data_Sheet_1.DOCX]

***Supplementary Material***

**Intra-muscular DNA vaccination against Spring Viraemia of Carp Virus induces full protection and establishes a viral-specific B and T cell response**

**Running title: DNA vaccination of carp against SVCV**

**Carmen W.E. Embregts, Dimitri Rigaudeau, Tomáš Veselý, Dagmar Pokorová, Niels Lorenzen, Jules Petit, Armel Houel, Malte Dauber, Heike Schütze, Pierre Boudinot, Geert F. Wiegertjes, Maria Forlenza^*^.**

*** correspondence: Maria Forlenza:** [**maria.forlenza@wur.nl**](mailto:maria.forlenza@wur.nl)

**A monoclonal raised against the SVCV G protein is able to specifically recognize the native G protein on infected and transfected EPC cells**

Antibodies were raised in mice by immunization with purified SVCV virus and the obtained supernatants were screened and validated through Western blot and immunofluorescence analyses of both infected and transfected EPC cells. Western blot analysis showed that clone 13C10c recognizes a single protein at around 90 kDa (Supplementary figure 1, A.2), which is higher than the calculated 57 kDa protein size of the SVCV G protein but is in accordance with the size detected using the previously validated anti-SVCV polyclonal rabbit antibodies (Hoffmann et al., 2002). This difference in size can be most likely ascribed to the presence of carbohydrate moieties on the glycoprotein. Next, infected and transfected EPC cells were used to show the ability of the antibody to also recognize the native SVCV G protein. EPCs were infected with SVCV at an MOI of 1 for 24h at 20°C and subsequently stained with the selected a-SVCV-G monoclonal antibody (13C10c). In parallel, EPC cells were transfected with pcDNA3-SVCV-G or pcDNA3 and were treated similarly. The antibody showed a strong reactivity to virus plaques in infected wells, but does not react with non-infected cells (Supplementary figure 1 B). In addition, a specific staining was observed in EPCs transfected with the pcDNA3-SVCV-G plasmid, but not in EPCs transfected with the pcDNA3 vector control (Supplementary figure 1C). Together this indicates that clone 13C10c is able to specifically recognize the native SVCV G protein and it is suitable for immunoblotting and immunohistochemical analysis.

**
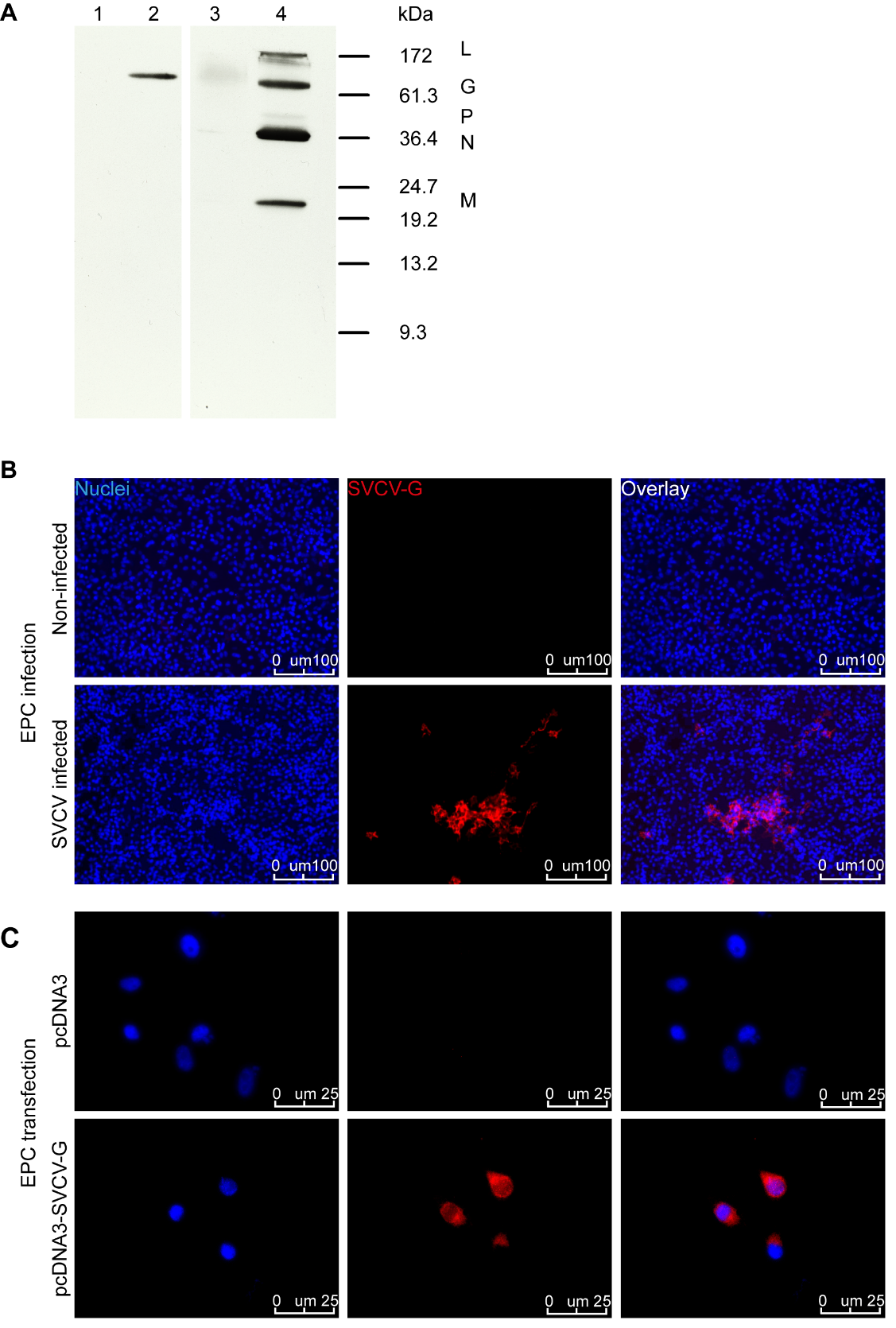
**

**Supplementary figure 1. Validation of a monoclonal antibody against the SVCV G protein. (A)** Proteins from non-infected EPCs (lanes 1 and 3) and purified virions (lanes 2 and 4) were resolved on a 15% SDS-PAGE gel. Proteins were visualized using hybridoma 13C10c supernatant (1:10; lanes 1-2) and anti-SVCV polyclonal rabbit serum (1:2000; lanes 3-4). **(B)** EPC cells were infected with SVCV (MOI=5) and imaged 24h later. **(C)** EPCs were transfected with 2 μg or pcDNA3-SVCV-G or pcDNA3 and imaged 48h later. Infected and transfected EPC were fixed, permeabilized and incubated with hybridoma 13C10c supernatant (1:150) followed by incubation with goat-anti-mouse RPE (1:500). DAPI (blue) counterstaining was used to visualize nuclei. Fluorescent images were acquired using a EVOS fl LED fluorescence microscope.
