# Supplementary figures and images for: Intramuscular DNA Vaccination of Juvenile Carp against Spring Viremia of Carp Virus Induces Full Protection and Establishes a Virus-Specific B and T Cell Response
Source: Front Immunol. 2017 Oct 24;8:1340. doi: 10.3389/fimmu.2017.01340 (PMC5660689; doi:10.3389/fimmu.2017.01340)

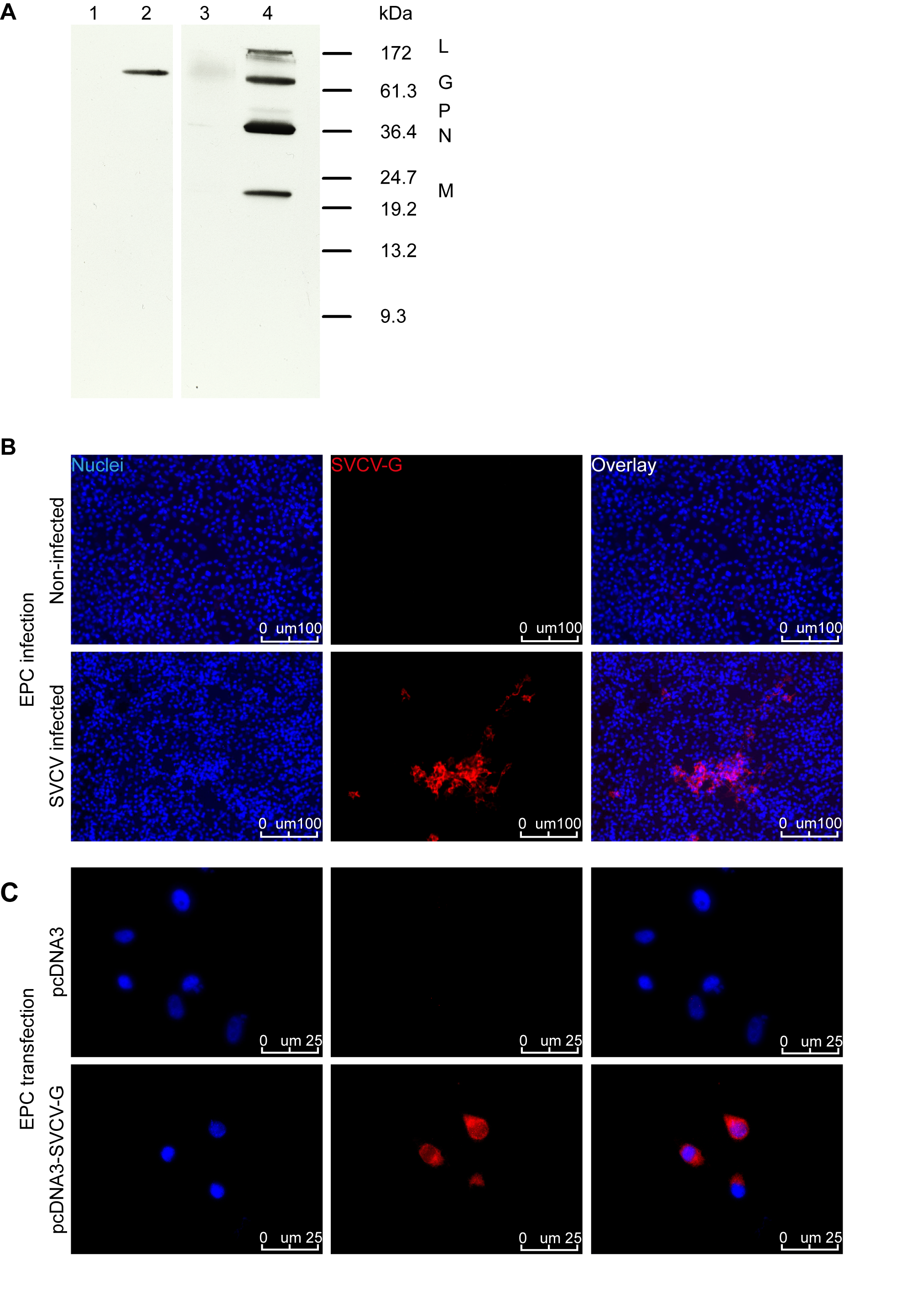

Supplement: Supplementary file 2 [file Image_1.TIF]
